# Diosgenin alleviates D-galactose-induced oxidative stress in rats’ brain and liver targeting aging and apoptotic marker genes

**DOI:** 10.3389/fmolb.2024.1303379

**Published:** 2024-02-23

**Authors:** Ali H. El-Far, Mona M. Elghaity, Shymaa A. Mohamed, Ahmed E. Noreldin, Yaser H. A. Elewa, Soad Khalil Al Jaouni, Abdelwahab A. Alsenosy

**Affiliations:** ^1^ Department of Biochemistry, Faculty of Veterinary Medicine, Damanhour University, Damanhour, Egypt; ^2^ Molecular Biology Unit, Medical Technology Centre, Medical Research Institute, Alexandria University, Alexandria, Egypt; ^3^ Histology and Cytology Department, Faculty of Veterinary Medicine, Damanhour University, Damanhour, Egypt; ^4^ Department of Histology, Faculty of Veterinary Medicine, Zagazig University, Zagazig, Egypt; ^5^ Faculty of Veterinary Medicine, Basic Veterinary Sciences, Hokkaido University, Sapporo, Japan; ^6^ Department of Hematology/Pediatric Oncology, Yousef Abdulatif Jameel Scientific Chair of Prophetic Medicine Application, Faculty of Medicine, King Abdulaziz University, Jeddah, Saudi Arabia

**Keywords:** diosgenin, oxidative stress, aging, antioxidants, brain, liver

## Abstract

The theory of aging is primarily concerned with oxidative stress caused by an imbalance in reactive oxygen species generation and cellular antioxidants. To alleviate the oxidative stress, we investigated the protective effect of diosgenin (DSG) for D-galactose (D-gal) using 20 and 40 mg of DSG/kg/day/orally for 42 days. The findings showed that D-gal caused brain and liver oxidative injuries by upregulating aging and oxidative markers. To counteract the oxidative stress caused by D-gal, DSG upregulated glutathione peroxidase-1, superoxide dismutase-1, and glutathione S-transferase-α. DSG also diminished the expression of *p53*, *p21*, Bcl-2-associated X protein, caspase-3, and mammalian target of rapamycin in brain and liver, as well as the build-up of *β*-galactosidase. DSG, in a dose-dependent manner, decreased the oxidative aging effects of D-gal in brain and liver tissues through targeting of aging and apoptotic marker genes. Finally, it should be noted that consuming DSG supplements is a suggesting natural preventative agent that may counteract aging and preserve health through improvement of body antioxidant status and control aging associated inflammation and cellular apoptosis.

## 1 Introduction

Aging is a complex biological process of gradually deteriorating an organism’s physiological functions over time (Rodríguez-Rodero et al., 2011). Aging is a progressive decline in cellular repair, leading to increased vulnerability to disease and, eventually, death (López-Otín et al., 2013). As we age at risk of severe cellular damage, two primary pathways contributing to oxidative stress and inflammation occur (Guzik and Touyz, 2017). Inflammaging is a hallmark of aging where the inflammatory molecules accumulate in cells, causing tissue damage, impairing organ function, and contributing to age-related illnesses (Ferrucci and Fabbri, 2018). Generation of reactive oxygen species (ROS) due to regular cellular metabolism can harm cellular DNA, proteins, and lipids. Over time, oxidative damage adds to cellular malfunction and aging (Juan et al., 2021). ROS are very reactive chemicals that can harm the activities of various cellular components (Birben et al., 2012).

D-galactose (D-gal)-induced aging is a model of accelerated aging in animals that involves administering D-gal to cause aging-like symptoms. This model is used in research to study the effects of aging and potential treatments for age-related conditions (Hou et al., 2019; de Almeida Rezende et al., 2021; Remigante et al., 2022; Wang et al., 2022). D-gal reacts with free amines of amino acids in proteins through nonenzymatic glycation to form advanced glycation end products. Also, chronic administration of D-gal could contribute to ROS generation through D-gal oxidative metabolism and glycation end products (Song et al., 1999; Parameshwaran et al., 2010).

D-gal-induced aging has been used to explore possible interventions like antioxidants, anti-inflammatory agents, and anti-aging compounds to mitigate the effects of aging (El-Far et al., 2020; El-Far et al., 2021; El-Far et al., 2022; de Almeida Rezende et al., 2021; Saafan et al., 2023). Due to their lack of addictive and poisonous properties, phytopharmaceuticals are becoming increasingly critical in allopathic and conventional medicine (Subhashini et al., 2011). Diosgenin (DSG) is a naturally occurring aglycone of the steroid saponin that is present in *Costus speciosus*, *Dioscorea* species *Smilax menispermoidea*, *Helicteres isora*, *Paris* species, *Aletris*, *Trigonella*, and *Trillium* (Zitka et al., 2012; Chiang et al., 2020). It has been tested pharmacologically for its ability to reduce blood sugar (McAnuff et al., 2005) and its anti-inflammatory and antioxidant potentials (Son et al., 2007; Al-Matubsi et al., 2011). Son et al. (2007) reported that, DSG increased total superoxide dismutase (T-SOD) glutathione peroxidase (GPx), and catalase in high-cholesterol fed rats. In addition, DSG significantly increased the antioxidative effect of dietary chromium chloride supplementation on high-cholesterol fed Japanese quails (Al-Matubsi et al., 2011). Besides, DSG inhibited the ER stress-induced inflammation in aorta in experimental diabetic rats (Prasad et al., 2022). The biological activities of DSG were stated in Supplementary Material S1 retrieved from the Comparative Toxicogenomics Database (CTD; http://ctdbase.org/), showing the preventive and curative roles of DSG in different diseases and the target genes. While DSG has been studied for its health benefits, its direct anti-aging effects are not well-established. Therefore, the current study was assigned to investigate the protective effects of DSG on D-gal induced aging in rats’ brains and liver through targeting of aging and apoptotic markers mRNA expressions.

## 2 Materials and methods

### 2.1 Ethical statement

The Faculty of Veterinary Medicine Ethics Committee at Damanhour University in Egypt has been accepted all techniques (DMU/VetMed-2023/028).

### 2.2 Animals and design

Fifty male Wistar rats were bought from the Medical Research Institute of Alexandria University in Egypt, weighing between 90 and 110 g B.W. Rats were received water and basal meal (Table 1) under controlled environmental conditions. Rats were housed 10 days before the trial for acclimatization.

**TABLE 1:** Ingredients of the basal diet.

Ingredients	g/kg diet
Corn flour	529.5
Casein	200
Sucrose	100
Soybean oil	70
Cellulose	50
Mineral mix	35
Vitamin mix	10
L-cystine	3
Choline	2.5

**SCHEME 1:**
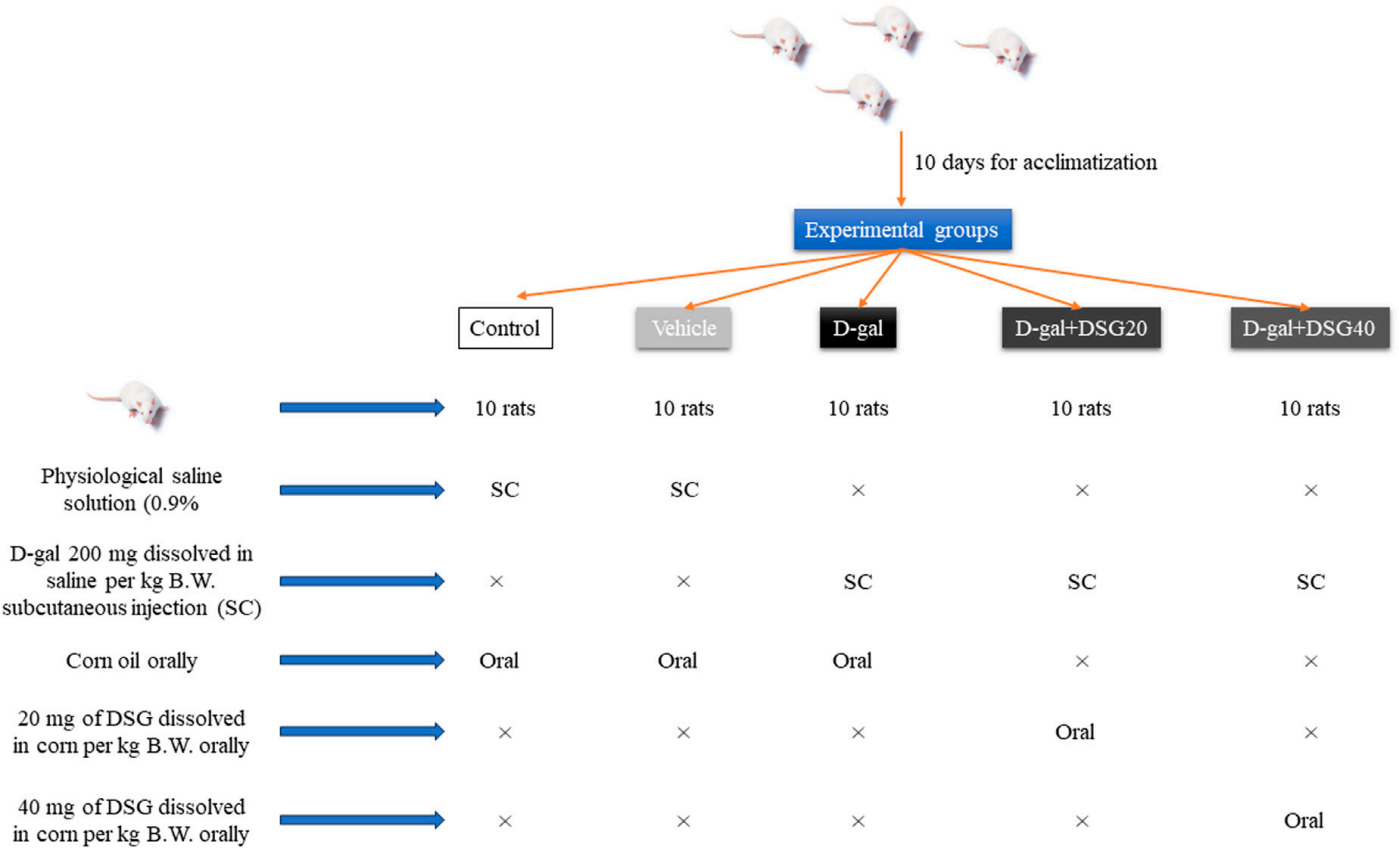
Experimental design.

Five groups of ten rats each were at random allotted into five equal groups (Scheme 1). Rats in the control group received daily subcutaneous injections of physiological saline solution (0.9%) for 42 days. Rats in the vehicle group received daily oral supplements of corn oil and saline injection subcutaneously for 42 days. Rats in the D-gal group received corn oil orally and daily subcutaneous injected with saline contains 200 mg of D-gal/kg body weight (B.W) (Fan et al., 2017; El-Far et al., 2021; 2022; Saafan et al., 2023) for 42 days. Rats in the D-gal + DSG20 group received 20 mg of DSG dissolved in corn per kg B.W. orally (Jagadeesan et al., 2012) and daily subcutaneous injected with saline contains 200 mg of D-gal/kg B.W. Rats in the D-gal + DSG40 group received 40 mg of DSG dissolved in corn per kg B.W. orally in addition to daily subcutaneous injected with saline contains 200 mg of D-gal/kg B.W. for 42 days.

### 2.3 Sampling

At the end of experiment, rats were inhaled isoflurane and cervical dislocation. The brains and livers were quickly rinsed with cold saline and cut into three pieces. One of the parts was used for histological study and was fixed in neutral buffered formaldehyde for 48 h, 10%, while the other two pieces were kept at −80°C for biochemical and mRNA expression analyses.

Caspase-3 (*CASP3*), B-cell lymphoma-2 (*Bcl2*), p53, p21, Bcl-2-associated X protein (*Bax*), mammalian target of rapamycin (*mTOR*), and β-actin were all expressed as mRNA in the first portion using RT-PCR. Malondialdehyde (MDA), superoxide dismutase 1 (SOD1), GPx-1, and glutathione S-transferase-α (GST-α) were all determined using ELISA in the second portion.

### 2.4 Histopathological assessment

The fixed brain and liver tissues were processed using the conventional paraffin embedding procedure. Then, 4 µm thick sections were stained by Hematoxylin and Eosin (H and E) (Bancroft and Layton, 2013). The brain and hepatic lesions were semi-quantitatively scored by examining 10 fields selected from each rat’s slide. The lesions were scored blindly as follows [Score scale: 0 = normal; 1 ≤ 25%; 2 = 26–50%; 3 = 51–75%; 4 = 76–100%] (Gibson-Corley et al., 2013).

### 2.5 Immunohistochemical assessment

Immunohistochemical assessment of *β*-galactosidase and 8-hydroxy-2′-deoxyguanosine (8-OHdG) was done following Noreldin et al. (2018) using antibodies listed in Table 2. The slices were photographed under a microscope (Leica DM500) using a digital camera (Leica EC3, Leica, Germany). We used the ImageJ software (National Institutes of Health, Bethesda, MD, United States) to quantify the immunostaining intensities according to Sysel et al. (2013). Furthermore, the inverse mean densities of 10 different fields from different sections were assessed according to Vis et al. (2000).

**TABLE 2:** Antibodies’ source, dilution, antigen retrieval, and heating condition.

Antibody	Source	Dilution	Antigen retrieval	Heating condition
Rabbit polyclonal anti- β-galactosidase	(PM049, MBL, WOBURN, MA, United States)	1:100	10 mM citrate buffer (pH 6.0)	105°C, 20 min
Mouse monoclonal anti-8-hydroxy-2′-deoxyguanosine (8-OHdG)	(ab48508, Abcam, Cambridge, United Kingdom)	1:200	10 mM citrate buffer (pH 6.0)	105°C, 20 min

### 2.6 Oxidative stress and antioxidants

Using chilled 0.1 M phosphate buffer saline and 20% (w/v), homogenates of the brain and liver were subjected to determination of MDA (Buege and Aust, 1978) and the protein levels of SOD1, GPx-1, and GST-*α* using ELISA kits (Fine Test, Wuhan, Hubei, China). All samples’ protein contents were assessed using the Bradford technique.

### 2.7 RNA extraction and real-time polymerase chain reaction (RT-PCR)

Total RNA was isolated from the tissue samples using the Easy spin kit for total RNA extraction following the manufacturer’s instructions (INTRON Biotechnology, Korea). Nanodrop spectrophotometer (Genway Nanodrop, Germany) was used to determine RNA’s purities and concentrations. Using the RT-Premix Kit, 1 µg of RNA was used to produce cDNA (INTRON Biotechnology). A final volume of 20 µL was created by combining 2 µL of RT product with 10 µL of SYBR-Green master mix, 0.5 mM of each forward and reverse primer (Table 3), and nuclease-free water. All reactions were done at 95°C for 10 min, then 40 cycles of 95°C for 15 s, 58°C for 15 s, and 72°C for 30 s using a 7,500 Applied Biosystems, United States. The housekeeper gene *β*-actin was used to standardize the relative expression of mRNA. The 2^−ΔΔCt^ approach, which Livak and Schmittgen (2001) developed, was used to calculate the fold changes in mRNA expression.

**TABLE 3:** Primer sequences for RT-PCR.

Genes	Primers 5^–^3	Accession number	Tm
*p53*	F: CCCACCATGAGCGTTGCT	NM_030989.3	60.36
	R: CCA​CCC​GGA​TAA​GAT​GTT​GG		62.46
*p21*	F: GAC​CTG​TTC​CAC​ACA​GGA​GCA​AAG	NM_080782.3	63.82
	R: GTC​TCA​GTG​GCG​AAG​TCA​AAG​TTC		62.07
*CASP3*	F: GAA​ATT​CAA​GGG​ACG​GGT​C	NM_012922.2	57.81
	R: TTC​TTT​GCA​TGG​AAA​GTG​GC		57.18
*Bax*	F: GCG​AAT​TGG​CGA​TGA​ACT​G	NM_017059.2	57.78
	R: ATG​GTT​CTG​ATC​AGC​TCG​G		56.92
*Bcl2*	_F:_ ACG​AGT​GGG​ATA​CTG​GAG​ATG​A	NM_016993.2	60.09
	R: TCT​CAG​GCT​GGA​AGG​AGA​AGA​T		60.29
*mTOR*	R: TCC​TGA​AGA​ACA​TGT​GCG​AG	NM_019906.2	57.92
	F: CCA​AAG​TAC​AAG​CGA​GAG​GC		58.92
*β-actin*	F: GCC​GTC​TTC​CCC​TCC​ATC​GTG	NM_031144.3	65.10
	R: TAC​GAC​CAG​AGG​CAT​ACA​GGG​ACA​AC		65.85

### 2.8 Molecular docking assessment

The three-dimension structures of RAC-alpha serine/threonine-protein kinase (AKT1), AKT2, AKT3, caspase-8, caspase-9, caspase-3, interleukin-6 receptor subunit alpha (IL6RA), IL6RB, mTOR, phosphatidylinositol 4,5-bisphosphate 3-kinase catalytic subunit alpha (PK3CA), and PK3CB were obtained from AlphaFold (https://alphafold.ebi.ac.uk/) protein structure database. Molecular Operating Environment (MOE 2015.10) software (Vilar et al., 2008) were used to prepare proteins for docking. In addition, the three-dimension structure of diosgenin was retrieved from PubChem (https://pubchem.ncbi.nlm.nih.gov/) database. Furthermore, MOE software did the molecular docking, protein-ligand interactions, and visualization.

### 2.9 Statistical analysis

GraphPad Prism v.9 (https://www.graphpad.com/) (GraphPad, San Diego, CA, United States) was used to analyze the data using a one-way ANOVA with Tukey’s *post hoc* multiple range testing. *p* < 0.05 was required for all significance declarations.

## 3 Results

### 3.1 Histopathology

**FIGURE 1:**
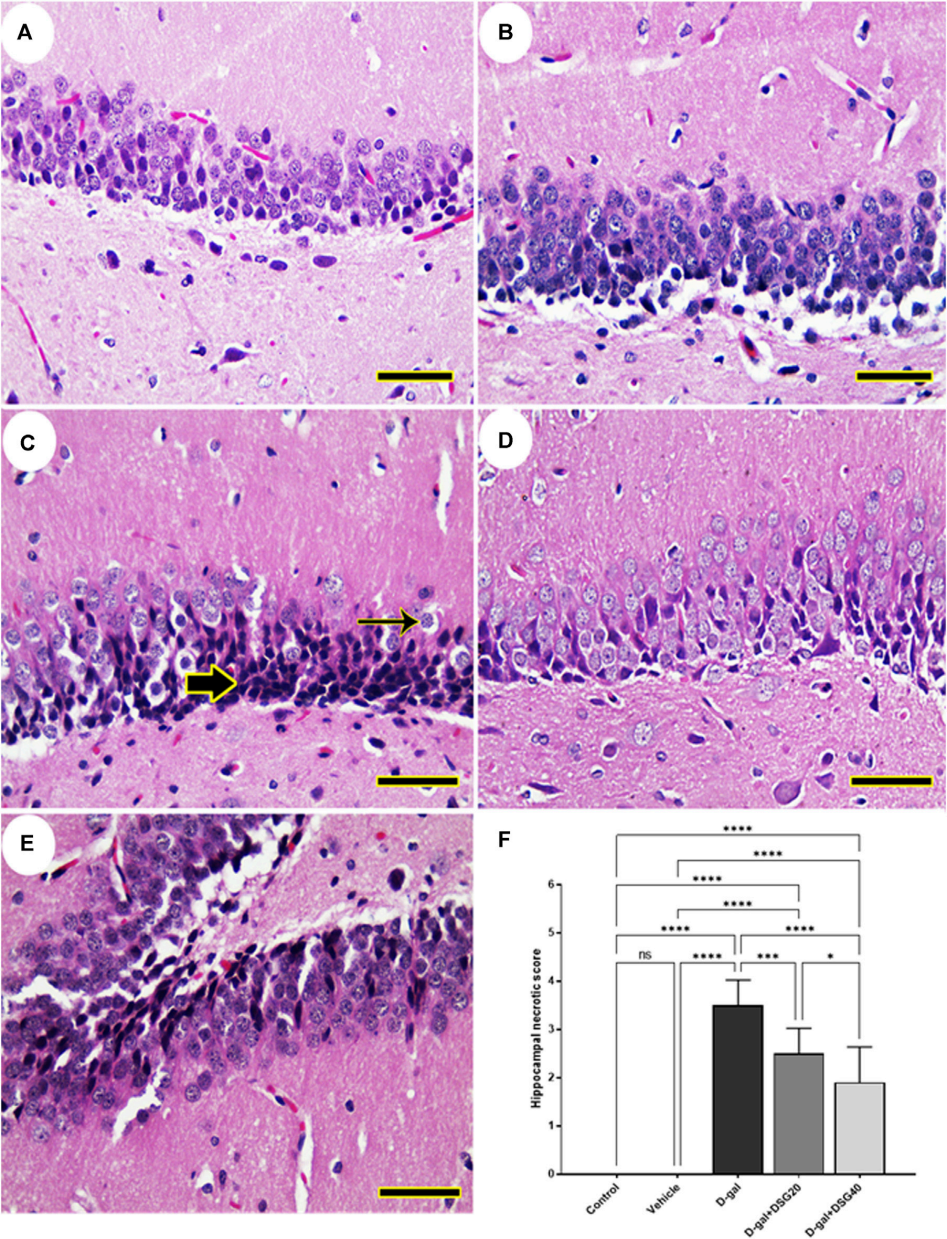
Representative photomicrograph of rat hippocampus. **(A)** Control group and **(B)** Vehicle group; rats showing normal histoarchitecture of the dentate gyrus. **(C)** D-gal-treated rats revealing shrunken neurons with pyknotic nuclei (thick arrow) and hyperchromatic neurons (thin arrow). **(D)** D-gal + DSG20-treated and **(E)** D-gal + DSG40-treated rats revealing improved hippocampal architecture. **(F)** Hippocampal necrotic scores. Data were analyzed with a one-way ANOVA followed by Tukey’s multiple comparison test. ns = nonsignificant, ^*^ *p* < 0.05, ^***^ *p* < 0.001, and ^****^ *p* < 0.0001. Error bars represent mean ± SD. (HE, *Scale bar= 50 µm*). D-gal; D-galactose. DSG; diosgenin.

The dentate gyrus was found to be normal in the control and vehicle groups’ hippocampus (Figures 1A, B). Nevertheless, necrotic neurons with pycnotic nuclei, hyperchromatic neurons, and very few normal neurons were recognized in the dentate gyrus of D-gal-treated rats (Figure 1C). In rats given D-gal + DSG20 and D-gal + DSG40, the dentate gyrus shape was enhanced, and there were fewer degenerating neurons (Figures 1D, E).

**FIGURE 2:**
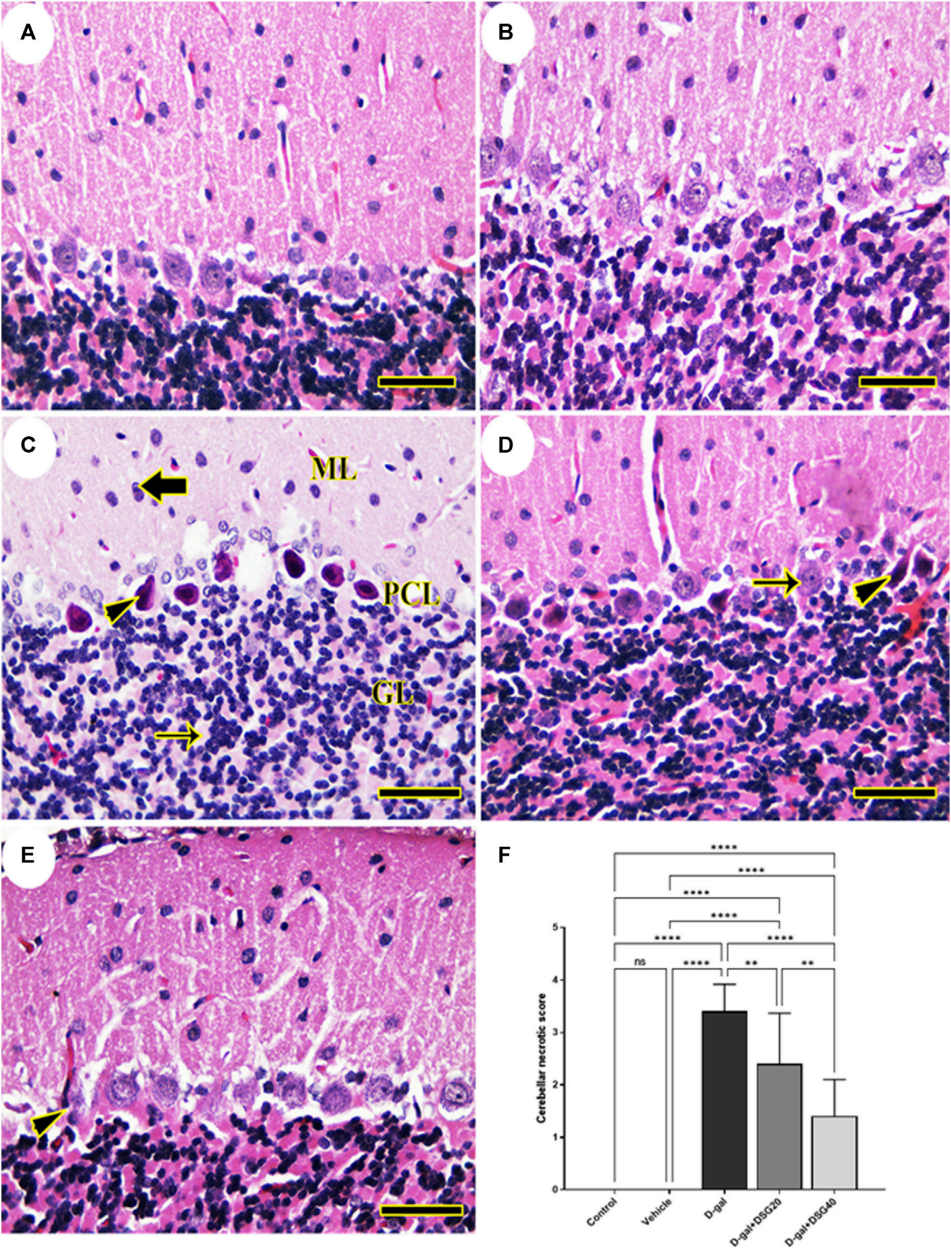
Representative photomicrograph of rat cerebellum. **(A)** Control group and **(B)** Vehicle group; rats illustrating normal histologic structure of cerebellum. **(C)** D-gal-treated rats revealing pyknotic Purkinje cells (arrowhead) in Purkinje cells layer (PCL), focal depletion of neurons (thin arrow) in granular layer (GL) and necrotic neurons (thick arrow) in molecular layer (ML). **(D)** D-gal + DSG20-treated rats showing nearly normal histologic structure with normal Purkinje cells (arrow) and a few lost or pyknotic (arrowhead) Purkinje cells. **(E)** D-gal + DSG40-treated rats showing normal hippocampal histoarchitecture with minimal degenerated (arrowhead) Purkinje cell. **(F)** Cerebellar necrotic scores. Data were analyzed with a one-way ANOVA followed by Tukey’s multiple comparison test. ns = nonsignificant, ^**^ *p* < 0.01, and ^****^ *p* < 0.0001. Error bars represent mean ± SD. (HE, *Scale bar = 50 µm*). D-gal; D-galactose. DSG; diosgenin.

We detected a typical cerebellar architecture comprising molecular layer, Purkinje cell, and granular layers when we examined the rats’ cerebella in the control and vehicle groups (Figures 2A, B). Rats given D-gal demonstrated focal loss of granular layer neurons and total loss of necrotic nuclei in the Purkinje cell layer (Figure 2C). However, the histologic structure of the cerebellum in the D-gal + DSG20-treated rats was better and had fewer pyknotic Purkinje cells (Figure 2D). Additionally, animals given D-gal + DSG40 treatment displayed virtually health cerebellar architecture with very few deteriorated Purkinje cells (Figure 2E).

**FIGURE 3:**
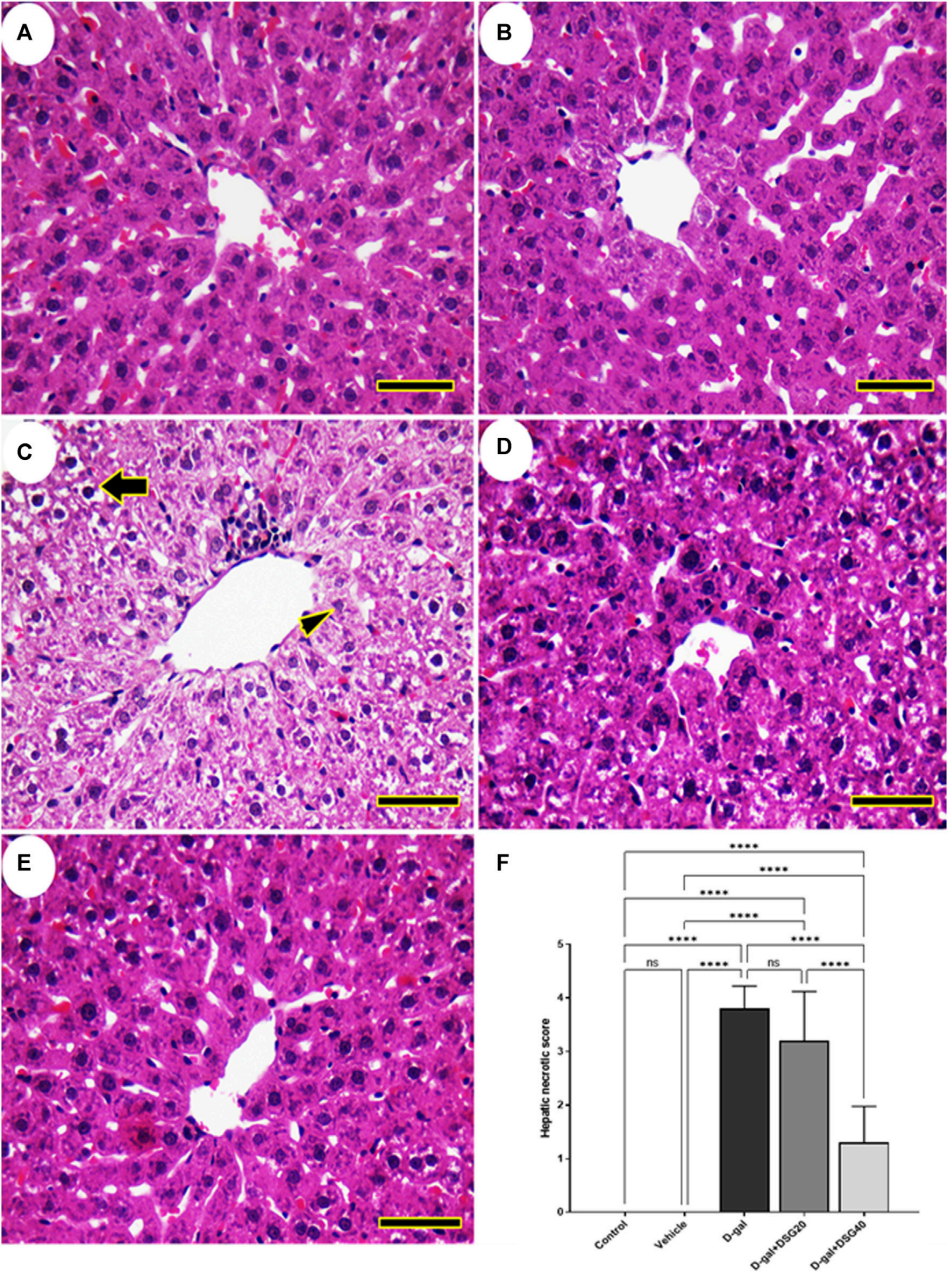
Representative photomicrograph of rat liver. **(A)** Control group. **(B)** vehicle group. **(C)** D-gal group showing hydropic degeneration (thick arrow) and necrosis of hepatocytes (arrowhead). **(D)** D-gal + DSG20. **(E)** D-gal + DSG40. **(F)** Hepatic necrotic scores. Data were analyzed with a one-way ANOVA followed by Tukey’s multiple comparison test. ns = nonsignificant. ^****^ *p* < 0.0001. Error bars represent mean ± SD. (HE, *Scale bar = 50 µm*). D-gal; D-galactose. DSG; diosgenin.

No histopathological hepatic abnormalities were found in the control and vehicle groups (Figures 3A, B). Conversely, the hepatic tissues of the D-gal group revealed hydropic degeneration, a build-up of inflammatory cells, and a dilated and blocked central vein (Figure 3C). The hepatic architecture improved in the D-gal + DSG20 group, and there were fewer pyknotic nuclei (Figure 3D). Additionally, compared with the control group, rats given D-gal + DSG40 treatment displayed a normal hepatic arch (Figure 3E). As represented in Figure 3F, the D-gal group exhibited a significantly higher vacuolation compared with the control group. On the other hand, hepatic lesions score significantly decreased with D-gal + DSG20 and D-gal + DSG40.

### 3.2 Immunohistochemistry

**FIGURE 4:**
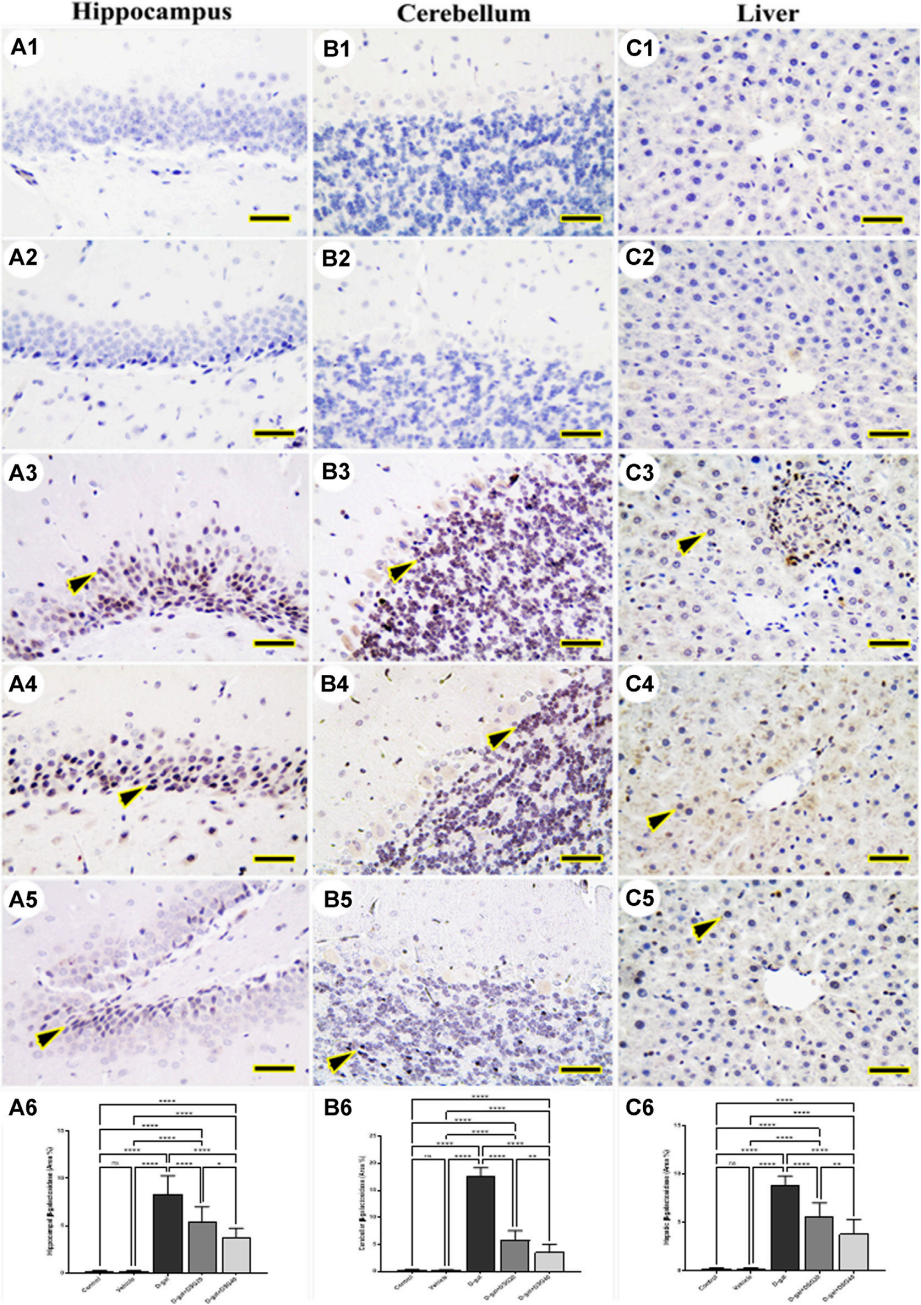
Representative photomicrograph demonstrated immunohistochemical expression of *β*-galactosidase in hippocampus **(A1–A5)**, cerebellum **(B1–B5)**, and liver **(C1–C5)** in control **(A1, B1, C1)**, vehicle **(A2, B2, C2)**, D-gal- treated **(A3, B3, C3)**, D-gal + DSG20-treated **(A4, B4, C4)**, and D-gal + DSG40-treated **(A5, B5, C5)**. Arrowheads indicate positive immune expression in D-gal-treated rats either with or without co-treatment with DSG20 or DSG40. **(A6)** Hippocampal *β*-galactosidase (Area %). **(B6)** Cerebellar *β*-galactosidase (Area %). **(C6)** Hepatic *β*-galactosidase (Area %). Data were analyzed with a one-way ANOVA followed by Tukey’s multiple comparison test. ns = nonsignificant, ^*^ *p* < 0.05, ^**^ *p* < 0.01, and ^****^ *p* < 0.0001. Error bars represent mean ± SD. *Scale bar* = 50 mm. D-gal; D-galactose. DSG; diosgenin.

**FIGURE 5:**
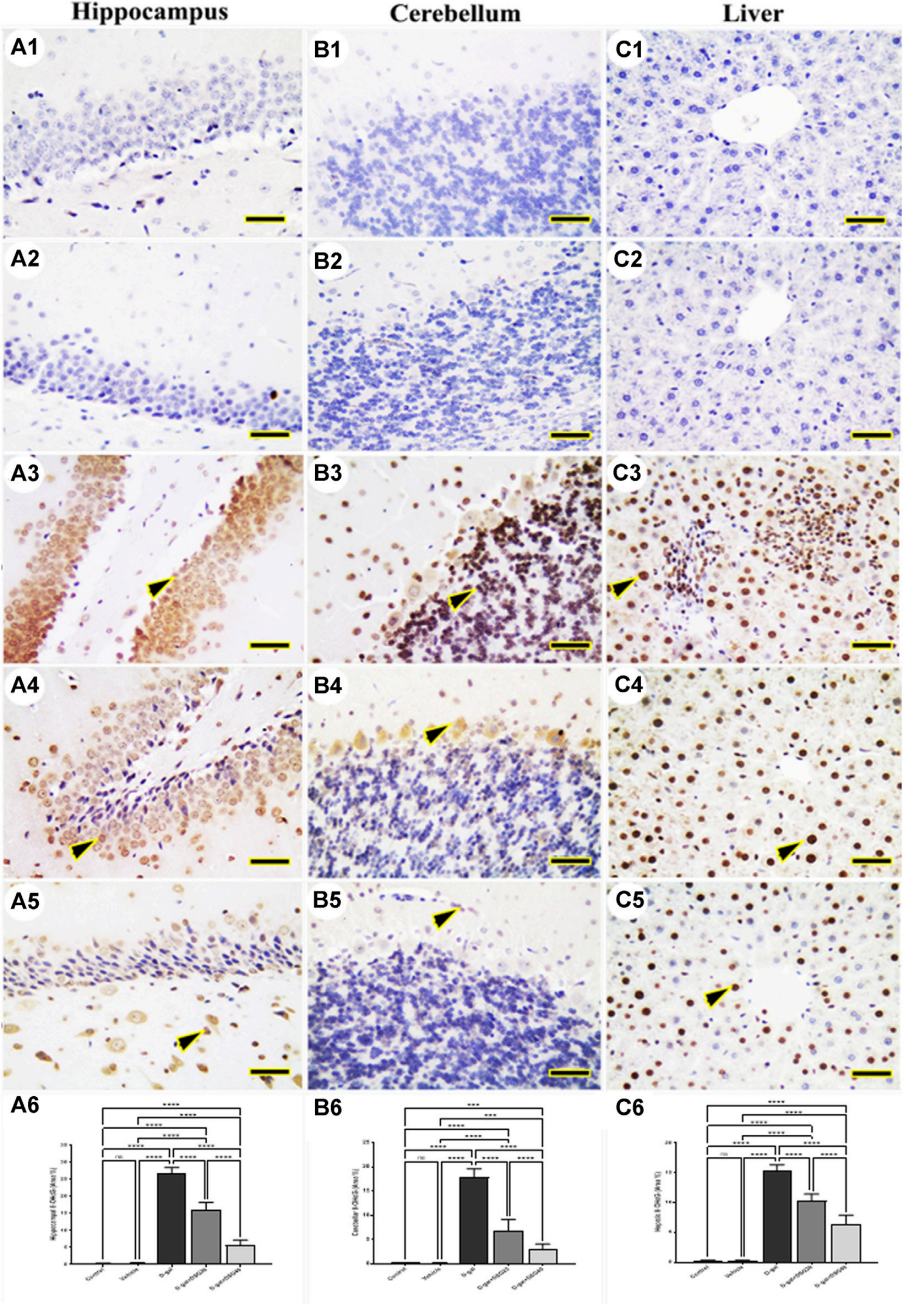
Representative photomicrograph demonstrated immunohistochemical expression of 8-hydroxy-2′-deoxyguanosine (8-OHdG) in hippocampus **(A1–A5)**, cerebellum **(B1–B5)**, and liver **(C1–C5)** from control **(A1, B1, C1)**, vehicle **(A2, B2, C2)**, D-gal-treated **(A3, B3, C3)**, D-gal + DSG20-treated **(A4, B4, C4)**, and D-gal + DSG40-treated **(A5, B5, C5)**. Arrowheads indicate positive immune expression in D-gal-treated rats either with or without co-treatment with DSG20 or DSG40. **(A6)** Hippocampal 8-OHdG (Area %). **(B6)** Cerebellar 8-OHdG (Area %). **(C6)** Hepatic8-OHdG (Area %). Data were analyzed with a one-way ANOVA followed by Tukey’s multiple comparison test. ns = nonsignificant, ^***^ *p* < 0.001, and ^****^ *p* < 0.0001. Error bars represent mean ± SD. *Scale bar* = 50 mm. D-gal; D-galactose. DSG; diosgenin.

The hippocampus (Figures 4A1, A2 and Figures 5A1, A2), cerebellum (Figures 4B1, B2 and Figures 5B1, B2), and liver (Figures 4C1, C2 and Figures 5C1, C2) revealed negative immune reactions for *β*-galactosidase and 8-OHdG, respectively in the control and vehicle groups. Conversely, the D-gal group displayed an abundance of *β*-galactosidase and 8-OHdG immune reactive nuclei in the brain regions (Figures 4A3, B3 and Figures 5A3, B3, respectively) and liver (Figure 4C3 and Figure 5C3, respectively).

In contrast, the D-gal group showed a higher distribution of *β*-galactosidase- and 8-OHdG-reacted nuclei than the D-gal + DSG20 group (Figures 4A4, B4, C4 and Figures 5A4, B4, C4, respectively). Additionally, the D-gal + DSG40 group showed the lowest distribution of nuclei reacting to *β*-galactosidase and 8-OHdG (Figures 4A5, B5, C5 and Figures 4A6, B6, C6, respectively). Rats treated with D-gal exhibit significantly higher expression than control rats in the area % of *β*-galactosidase and 8-OHdG’s immunohistochemical reacted nuclei. In the D-gal + DSG20 and D-gal + DSG40 groups, this expression was significantly reduced (Figures 4A6, B6, C6 and Figures 5A6, B6, C6, respectively).

### 3.3 Antioxidant status

**FIGURE 6:**
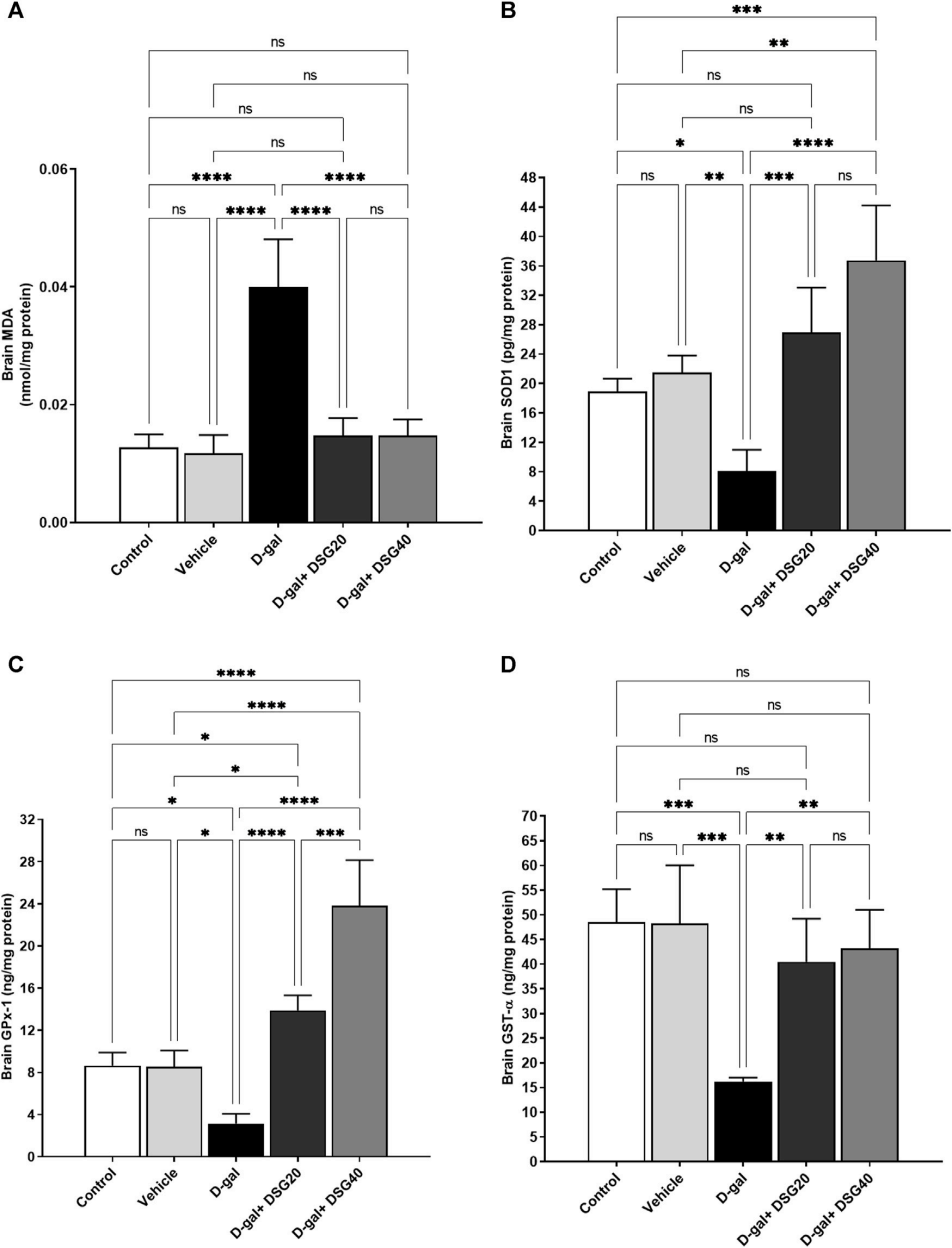
Brain oxidative stress and antioxidant status. **(A)** Brain malondialdehyde (MDA). **(B)** Brain superoxide dismutase (SOD1). **(C)** Brain glutathione peroxidase (GPx-1). **(D)** Brain glutathione S-transferase (GST-*α*). Data were analyzed with a one-way ANOVA followed by Tukey’s multiple comparison test. ns = nonsignificant, ^*^ *p* < 0.05, ^**^ *p* < 0.01, ^***^ *p* < 0.001, and ^****^ *p* < 0.0001. Error bars represent mean ± SD. *n* = 4. D-gal; D-galactose. DSG; diosgenin.

Regarding the data shown in Figure 6A, the brain MDA concentration increased significantly (*p* < 0.0001) in the D-gal group compared with the control and vehicle groups. While it decreased significantly (*p* < 0.0001) in the D-gal + DSG20 and D-gal + DSG40 groups. Compared with the control and vehicle groups, there was no apparent difference between the D-gal + DSG20 and D-gal + DSG40 groups.

According to the data in Figure 6B, brain SOD1 concentrations were significantly lower in the D-gal group than the control (*p* < 0.05) and vehicle groups (*p* < 0.01) and higher in the D-gal + DSG20 (*p* < 0.001) and D-gal + DSG40 groups (*p* < 0.0001) than in the D-gal group.

Its concentration was not changed considerably in the D-gal + DSG20 group relative to the control group, but it was significantly higher in the D-gal + DSG40 group (*p* < 0.001) than the control group.

According to the data in Figure 6C, the brain GPx-1 levels were significantly (*p* < 0.05) lower in the D-gal group than in the control and vehicle groups. In contrast, it was significantly (*p* < 0.0001) higher in the D-gal + DSG20 and D-gal + DSG40 groups than in the D-gal group. Additionally, its concentration was considerably higher in the D-gal + DSG40 group in comparison with the control, vehicle, and D-gal + DSG20 groups (*p* < 0.0001), (*p* < 0.0001), and (*p* < 0.001), respectively.

According to the data shown in Figure 6D, the brain GST-α levels were significantly (*p* < 0.001) lower in the D-gal group compared with the control and vehicle groups. At the same time, it was significantly (*p* < 0.01) higher in the D-gal + DSG20 and D-gal + DSG40 groups compared with the D-gal group. Furthermore, compared with the control and vehicle groups, there was no considerable difference in their concentration in the D-gal + DSG20 and D-gal + DSG40 groups.

**FIGURE 7:**
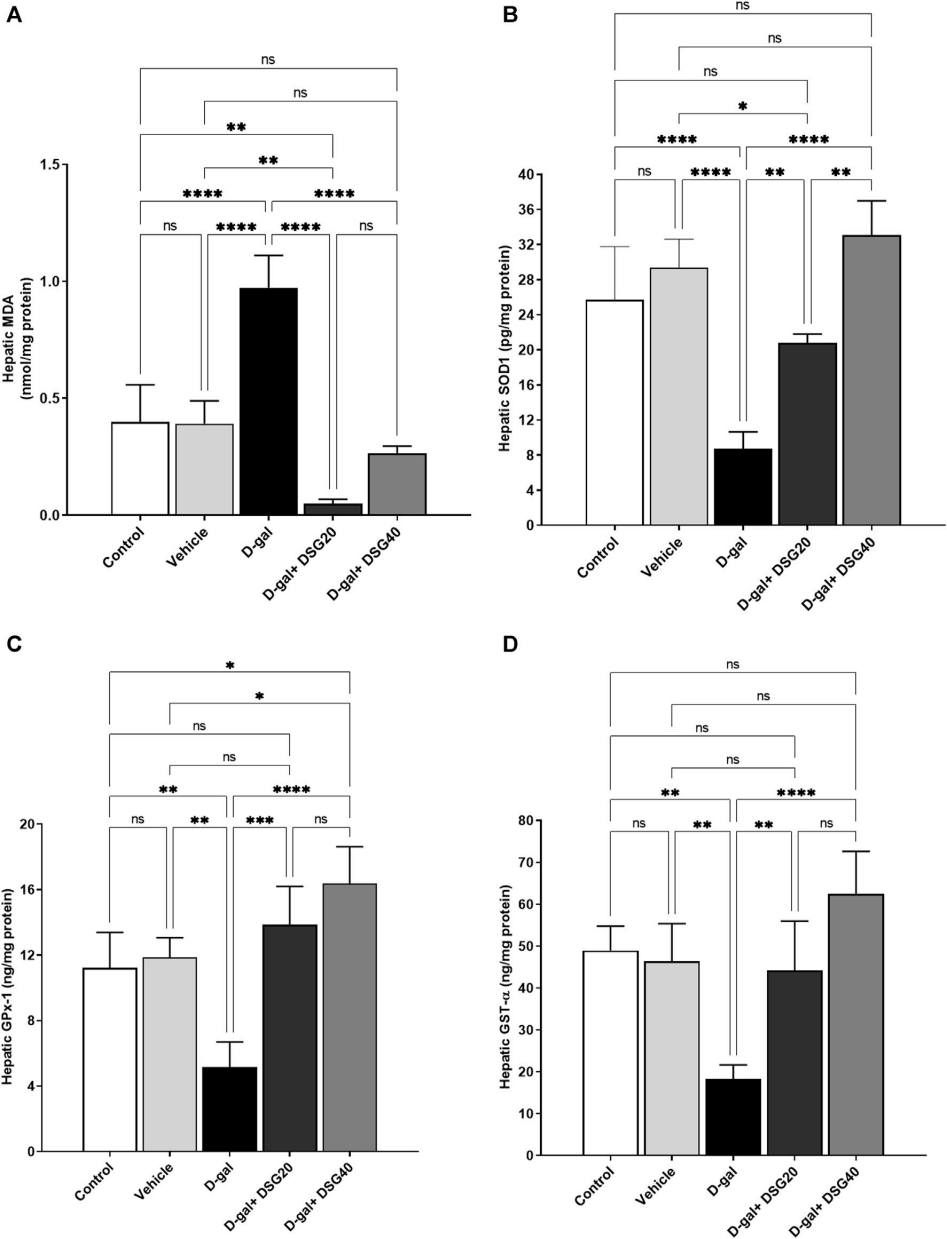
Hepatic oxidative stress and antioxidant status. **(A)** Hepatic MDA. **(B)** Hepatic SOD1. **(C)** Hepatic GPx-1. **(D)** Hepatic GST-*α*. Data were analyzed with a one-way ANOVA followed by Tukey’s multiple comparison test. ns = nonsignificant, ^*^ *p* < 0.05, ^**^ *p* < 0.01, ^***^ *p* < 0.001, and ^****^ *p* < 0.0001. Error bars represent mean ± SD. *n* = 4. D-gal; D-galactose. DSG; diosgenin.

MDA levels were higher (*p* < 0.0001) in the D-gal group (Figure 7A). SOD1 (Figure 7B), GPx-1 (Figure 7C), and GST-α (Figure 7D) levels were all considerably lower (*p* < 0.0001, *p* < 0.01, and *p* < 0.01, respectively). The D-gal treated with DSG demonstrated dose-dependently substantial increases in SOD1, GPx-1, and GST-α and significant decreases in MDA in the D-gal + DSG20 and D-gal + DSG40 groups (*p* < 0.0001).

### 3.4 mRNA expression

**FIGURE 8:**
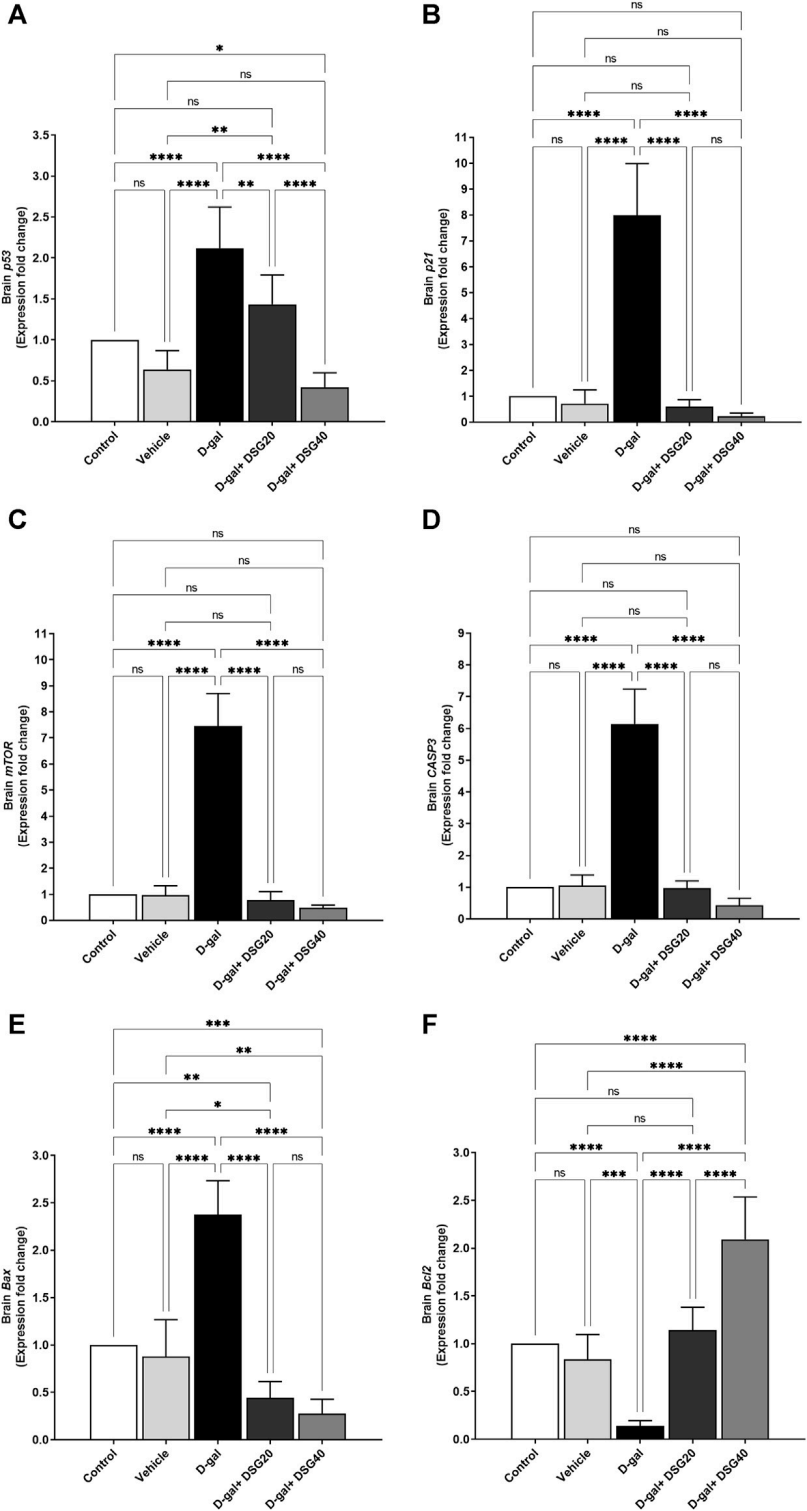
mRNA relative fold change expression of brain tissue. **(A)** *p53*. **(B)** *p21*. **(C)** Mammalian target of rapamycin (*mTOR*). **(D)** caspase-3 (*CASP3*). **(E)** Bcl2-associated X protein (*Bax*). **(F)** And B-cell lymphoma 2 (*Bcl2*). Data were analyzed with a one-way ANOVA followed by Tukey’s multiple comparison test. ns = nonsignificant, ^*^ *p* < 0.05, ^**^ *p* < 0.01, ^***^ *p* < 0.001, and ^****^ *p* < 0.0001. Error bars represent mean ± SD. *n* = 6. D-gal; D-galactose. DSG; diosgenin.

Relative to the control and vehicle groups, the brain’s *p53* mRNA levels in the D-gal group were considerably higher (*p* < 0.0001). *P53* expressions were significantly lower in the D-gal + DSG20 (*p* < 0.01) and D-gal + DSG40 (*p* < 0.0001) groups compared with the D-gal group. Also, compared with the control (*p* < 0.05) and D-gal + DSG20 groups, its expression levels were considerably lower in the D-gal + DSG40 group (Figure 8A).

Related to the control and vehicle groups, the brain *p21* mRNA expressions in the D-gal group were considerably higher (*p* < 0.0001). However, as compared with the D-gal group, the brain p21 mRNA expressions in the D-gal + DSG20 and D-gal + DSG40 groups were significantly lower (*p* < 0.0001) than in the D-gal group (Figure 8B).

Relative to the control and vehicle groups, the brain *mTOR* mRNA expressions in the D-gal group were considerably higher (*p* < 0.0001). However, when compared with the D-gal group, its expressions were dramatically reduced (*p* < 0.0001) in the D-gal + DSG20 and D-gal + DSG40 groups (Figure 8C).

Compared with the control and vehicle groups, the brain *CASP3* mRNA expressions in the D-gal group were considerably higher (*p* < 0.0001). Figure 8D shows that the levels in the D-gal + DSG20 and D-gal + DSG40 groups were significantly lower (*p* < 0.0001) than those in the D-gal group.

Compared with the control and vehicle groups, the brain *Bax* mRNA expressions in the D-gal group were considerably higher (*p* < 0.0001). At the same time, the brain *Bax* mRNA expressions in the D-gal + DSG20 and D-gal + DSG40 groups were significantly lower (*p* < 0.0001) than those in the D-gal group. The brain *Bax* mRNA expressions were considerably down in the D-gal + DSG20 (*p* < 0.01) and D-gal + DSG40 (*p* < 0.001) than the control group (Figure 8E).

When compared with the control and vehicle groups, the brain *Bcl2* mRNA levels in the D-gal group were considerably lower (*p* < 0.0001) and lower (*p* < 0.001). On the other hand, relative to the D-gal group, the *Bcl2* mRNA expressions in the brain were significantly higher in the D-gal + DSG20 and D-gal + DSG40 groups (*p* < 0.0001). Additionally, compared with the control, vehicle, and D-gal + DSG20 groups, *Bcl2* expressions were considerably (*p* < 0.0001) higher in the D-gal + DSG40 group (Figure 8F).

**FIGURE 9:**
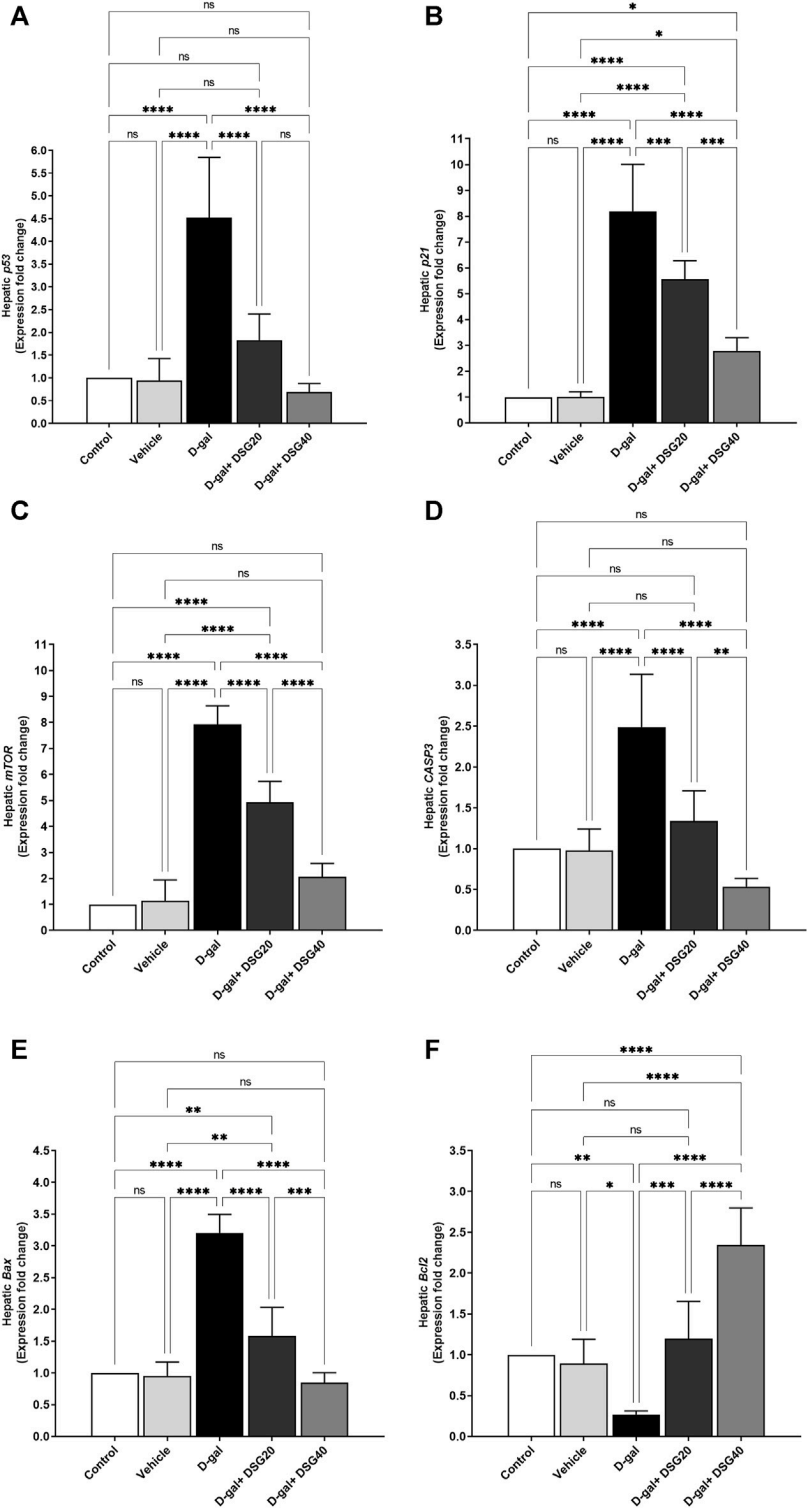
mRNA relative fold change expression of hepatic tissue. **(A)** *p53*. **(B)** *p21*. **(C)** Mammalian target of rapamycin (*mTOR*). **(D)** caspase-3 (*CASP3*). **(E)** Bcl2-associated X protein (*Bax*). **(F)** And B-cell lymphoma 2 (*Bcl2*). Data were analyzed with a one-way ANOVA followed by Tukey’s multiple comparison test. ns = nonsignificant, ^*^ *p* < 0.05, ^**^ *p* < 0.01, ^***^ *p* < 0.001, and ^****^ *p* < 0.0001. Error bars represent mean ± SD. *n* = 6. D-gal; D-galactose. DSG; diosgenin.

Like how *Bcl2* (Figure 9F) expression was dramatically reduced (*p* < 0.01), hepatic *p53* (Figure 9A), *p21* (Figure 9B), *mTOR* (Figure 9C), *CASP3* (Figure 9D), and *Bax* (Figure 9E) mRNA expression was significantly elevated in the D-gal group (*p* < 0.0001) compared with control. In contrast, *Bcl2* expression was dramatically elevated in a dosage-dependent manner in the D-gal + DSG20 and D-gal + DSG40 groups than the D-gal group, while *p53, p21, CASP3, Bax,* and *mTOR* mRNA levels were significantly lowered.

### 3.5 Molecular docking

**FIGURE 10:**
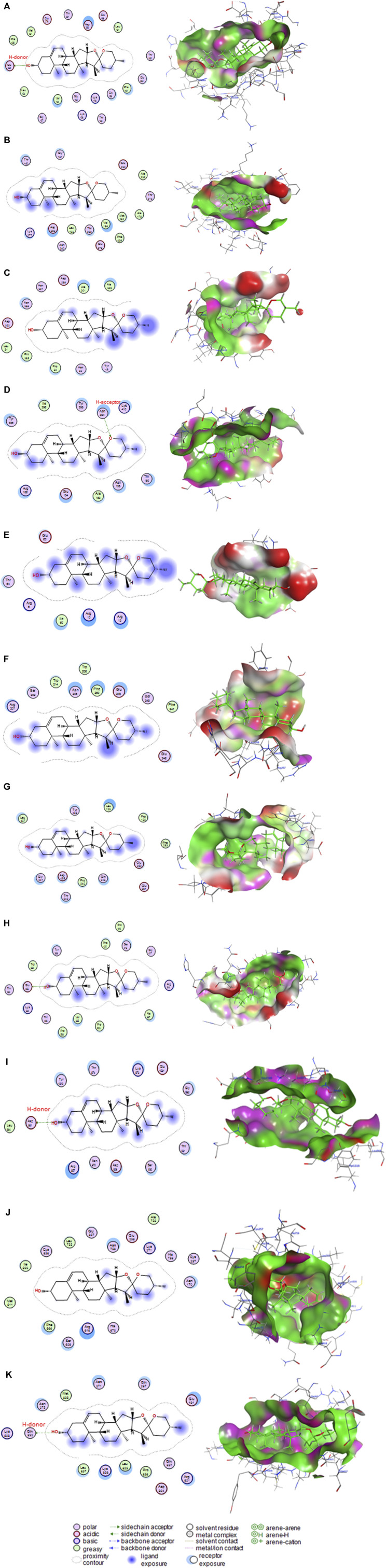
Molecular docking interaction of diosgenin with **(A)** protein kinase B1 (AKT1), **(B)** protein kinase B2 (AKT2), **(C)** protein kinase B3 (AKT3), **(D)** caspase-8, **(E)** caspase-9, **(F)** caspase-3, **(G)** interleukin-6 receptor subunit alpha (IL6RA), **(H)** interleukin-6 receptor subunit beta (IL6RB), **(I)** mammalian target of rapamycin (mTOR), **(J)** phosphatidylinositol 4,5-bisphosphate 3-kinase catalytic subunit alpha (PK3CA), and **(K)** phosphatidylinositol 4,5-bisphosphate 3-kinase catalytic subunit beta (PK3CB).

Diosgenin interacted with the binding sites of rats’ AKT1 (Figure 10A), AKT2 (Figure 10B), AKT3 (Figure 10C), caspase-8 (Figure 10D), caspase-9 (Figure 10E), caspase-3 (Figure 10F), IL6RA (Figure 10G), IL6RB (Figure 10H), mTOR (Figure 10I), PK3CA (Figure 10J), and PK3CB (Figure 10J) by binding energies of −6.68, −6.26, −5.79, −7.06, −4.66, −5.72, −5.78, −7.02, −5.75, −6.71, and −6.77 kcal/mol, respectively.

## 4 Discussion

Cellular hallmarks of aging include accumulation of *β*-galactosidase and overexpression of *p53* and *p21*. Expression levels of *p53* and *p21* mRNA were upregulated along with considerable *β*-galactosidase immunostaining expression in the D-gal group. El-Far et al. (2021, 2022) and Saafan et al. (2023) found that rats in the D-gal groups had significantly higher levels of *p53* and *p21* expression in their brains. Additionally, mice given D-gal injections showed an increase in brain p21, according to Sun et al. (2018). Further, p53 and p21 were upregulated in the pancreas and kidneys of rats that had received D-gal injections to accelerate aging (El-Far et al., 2020). Western blot research has shown that rats given D-gal treatment have considerably higher p53, p21, and *β*-galactosidase protein expression in their liver (Huang et al., 2013). Furthermore, a significant build-up of *β*-galactosidase in the aged rats’ hippocampus (Li et al., 2016).

Neurodegenerative disorders and the natural aging process have oxidative stress as a significant contributing element (Yin et al., 2009). Increased ROS levels are likely to trigger cellular senescence (Liguori et al., 2018). However, the precise mechanism of oxidative stress-induced aging is uncertain. In the current investigation, D-gal markedly reduced SOD1, GPx-1, and GST levels in the brain and liver. Similarly, significant increases in the expression of 8-OHdG levels were reported by Du et al. (2019). The authors recognized reduction in total SOD and GPx activities in the D-gal-induced aging paradigm. Similar to this, animals treated with D-gal had their brain GPx and GST activities significantly reduced (El-Far et al., 2022). Also, SOD1 protein (Kuo et al., 2022) and SOD activity (Kuo et al., 2022) were decreased in the brain of rats injected with D-gal. Significant drops in the levels of Sirt1, Bcl2, CAT, and GPx were reported by Motevalian et al. (2021) in D-gal-treated mice.

In the present study, DSG overcame the oxidative and apoptotic alterations induced by D-gal in the brain and hepatic tissues. Moreover, molecular docking assessment stated the binding affinity of DSG to control apoptotic and inflammatory targets. By lowering a build-up, increasing SOD activity, and minimizing lipid peroxidation, and DSG prevented brain cell death. Similarly, Koh et al. (2016) reported significant enhancement in SOD activity with reduction in MDA levels due to DSG in the brain with neuronal damage induced by Aβ-42 accumulation and neurotoxicant injection. Also, DSG suppressed D-gal-induced neuronal Fas-dependent and mitochondria-dependent apoptotic pathways in rats (Cheng et al., 2020). Moreover, DSG successfully protected the dopaminergic neurons from LPS-induced neuroinflammation that was monitored by significant reduction in tumor necrosis factor-α and inducible nitric oxide synthase (Lee et al., 2021).

Free radicals produced lipid peroxidation, deactivation of enzymes, apoptosis and DNA breakup (Valko et al., 2007). In the current investigation, we reported significant reductions in *Bcl2* expression and increased apoptosis, as evidenced by overexpression of *CASP3* and *Bax* expression, in the hippocampus of rats. Atef et al. (2022) found increased immunostaining expression caspase-3 in the brain. Moreover, caspase-3, Bcl2, Bax, and *CASP3* levels in the brain and heart were also increased in rats treated with D-gal (El-Far et al., 2021). Similarly, in our previous study, we reported significant reductions in Bcl2 expression and increased apoptosis as evidenced by overexpression of *CASP3*, *Bax*, and Bax protein (El-Far et al., 2021).

Natural compounds have been extensively employed in D-gal-inducing models for their anti-aging properties (El-Far et al., 2020; El-Far et al., 2021; El-Far et al., 2022; Saafan et al., 2023). The current investigation dramatically improved the antioxidant status of the rats’ brains and livers by adding DSG to the D-gal-treated rats. In the same setting, D-gal significantly raised the Bax/Bcl-2 ratio and caspase-3 in the brains of mice (Zhang et al., 2019) and rats (Ullah et al., 2015). On the contrary, DSG may have neuroprotective effects for preventing D-gal-induced brain aging by enhancing the *Bcl*-2 family (Cheng et al., 2020). Also, the mTOR is known to control various signs of aging (Weichhart, 2018). We reported a substantial overexpression of mTOR in the current investigation. Similarly, mTOR expression was elevated due to D-gal (Saafan et al., 2023).

## 5 Conclusion

Inflammation and oxidative stress are recognized as the two primary aging-related processes. Through the downregulation of aging markers (*p53, p21*, and *β*-galactosidase) and apoptotic markers (*Bax* and *CASP3*), as well as the improvement of antioxidant status, histomorphology, and immunohistochemical evaluation of the brain and hepatic tissues, DSG may have been able to mitigate the oxidative stress caused by D-gal in the rat brain and liver tissues. Besides, DSG exhibited a high affinity to inhibit apoptotic and inflammatory target proteins. Our findings imply that DSG successfully slowed down the rats’ brain and liver tissue aging through targeting of aging and apoptotic genes along with enhancement of cellular antioxidant status and overcome of inflammatory process, making it a potentially effective natural anti-aging supplement.

## Data Availability

The raw data supporting the conclusions of this article will be made available by the authors, without undue reservation.
